# Upregulation of Phagocyte-Derived Catecholamines Augments the Acute Inflammatory Response

**DOI:** 10.1371/journal.pone.0004414

**Published:** 2009-02-12

**Authors:** Michael A. Flierl, Daniel Rittirsch, Brian A. Nadeau, J. Vidya Sarma, Danielle E. Day, Alex B. Lentsch, Markus S. Huber-Lang, Peter A. Ward

**Affiliations:** 1 Department of Pathology, University of Michigan Medical School, Ann Arbor, Michigan, United States of America; 2 The Laboratory of Trauma, Sepsis & Inflammation Research, Department of Surgery, University of Cincinnati College of Medicine, Cincinnati, Ohio, United States of America; 3 Department of Trauma-, Hand- and Reconstructive Surgery, University of Ulm Medical School, Ulm, Germany; Instituto Oswaldo Cruz and FIOCRUZ, Brazil

## Abstract

Following our recent report that phagocytic cells (neutrophils, PMNs, and macrophages) are newly discovered sources of catecholamines, we now show that both epinephrine and norepinephrine directly activate NFκB in macrophages, causing enhanced release of proinflammatory cytokines (TNFα, IL-1β, IL-6). Both adrenal-intact (AD+) and adrenalectomized (ADX) rodents were used, because ADX animals had greatly enhanced catecholamine release from phagocytes, facilitating our efforts to understand the role of catecholamines released from phagocytes. Phagocytes isolated from adrenalectomized rats displayed enhanced expression of tyrosine-hydroxylase and dopamine-β-hydroxylase, two key enzymes for catecholamine production and exhibited higher baseline secretion of norepinephrine and epinephrine. The effects of upregulation of phagocyte-derived catecholamines were investigated in two models of acute lung injury (ALI). Increased levels of phagocyte-derived catecholamines were associated with intensification of the acute inflammatory response, as assessed by increased plasma leak of albumin, enhanced myeloperoxidase content in lungs, augmented levels of proinflammatory mediators in bronchoalveolar lavage fluids, and elevated expression of pulmonary ICAM-1 and VCAM-1. In adrenalectomized rats, development of ALI was enhanced and related to α_2_-adrenoceptors engagement but not to involvement of mineralocorticoid or glucocorticoid receptors. Collectively, these data demonstrate that catecholamines are potent inflammatory activators of macrophages, upregulating NFκB and further downstream cytokine production of these cells. In adrenalectomized animals, which have been used to further assess the role of catecholamines, there appears to be a compensatory increase in catecholamine generating enzymes and catecholamines in macrophages, resulting in amplification of the acute inflammatory response via engagement of α_2_-adrenoceptors.

## Introduction

During an immune response, the central nervous system and the immune system communicate with each other [1]. The major pathway systems involved in this cross-talk are the hypothalamic-pituitary-adrenal (HPA) axis and the autonomic nervous system [1]–[3]. Activation of the vagus-dominated parasympathetic, cholinergic nervous system is known to greatly attenuate and dampen the inflammatory response via nicotinergic cholinergic receptors expressed on macrophages and other immune cells [4], [5]. According to its afferent and efferent arms, this effect has been termed “inflammatory reflex” [2] or “cholinergic anti-inflammatory pathway” [6]. In contrast, the role of the sympathetic nervous system (SNS) during inflammation seems to be more complex and less well understood. On the one hand, SNS activation seems to target immune cells that express adrenoreceptors, exacerbating the local inflammatory response [7], [8], and increase the general immune and proinflammatory mediator response [9]–[11]. On the other hand, several studies indicate an inhibitory effect of the SNS on the inflammatory response, suppressing the immune response by decreasing the activity of natural killer cells and T cell immunity [12]–[15]. In addition, catecholamines released from presynaptic sympathetic nerve terminals lead to localized vasoconstriction, preventing invading pathogens from becoming systemic [3].

Over two decades ago, lymphocytes were described as sources of catecholamines [16]. These lymphocyte-derived catecholamines seem to act in an autocrine/paracrine fashion that affects lymphocyte trafficking [17], vascular perfusion, cell proliferation [18], cytokine production and the functional activity of lymphocytes [19], [20]. Recently, phagocytes (macrophages and neutrophils) have also been identified as a newly recognized source of catecholamines that exert a similar autocrine/paracrine regulation of phagocytes following release of norepinephrine or epinephrine [8], [20]–[22]. Additional experiments demonstrated that blockade of these phagocyte-derived catecholamines (by pharmacological blockade of catecholamine generating enzymes or blockade of adrenoceptors) greatly attenuated lung inflammatory injury, while the opposite was the case when the catecholamine-inactivating enzymes catechol-*O*-methyltransferase (COMT) and monoamine oxidase (MAO) were inhibited [8]. Therefore, activation of the adrenergic system during an inflammatory response may greatly enhance the local inflammatory response, resulting in neutrophil accumulation [23] and enhanced cytokine production. In the current study, we sought to further define the role of phagocyte-derived catecholamines on inflammation on a molecular level and in a setting of acute inflammatory, single organ injury.

## Results

### Catecholamines Induce NFκB Activation and Release of Cytokines in Mouse Macrophages

**Figure 1:**
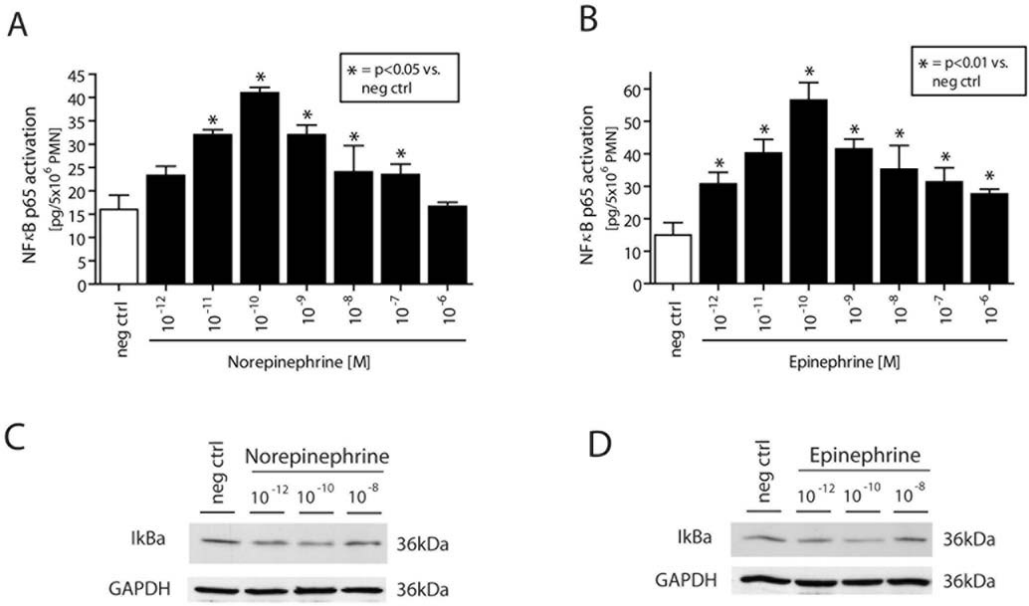
Isolated peritoneal mouse macrophages (A, B) were exposed to various concentrations of norepinephrine or epinephrine for 30 min at 37°C. Then, nuclear proteins were extracted from 10×10^6^ cells protein concentrations adjusted and NFκB p65 activation assessed. Each bar represents n = 4. In a second set of experiments, following incubation with various concentrations of norepinephrine or epinephrine (30 min, 37°C), peritoneal mouse macrophages (5×10^6^/ml; C, D) were lysed and cytosol subjected to Western blotting analysis for IκBα. Depicted blots are representative of 3 independent experiments. Neg ctrl, incubation with HBSS.

Following isolation, peritoneal mouse macrophages were stimulated with a range of concentrations of norepinephrine or epinephrine (10^−12^–10^−6^ M) for 30 min at 37°C. While unstimulated macrophages exhibited low levels of NFκB activation, significant NFκB activation occurred in a dose-dependent manner, peaking at the 10^−10^ M dose of catecholamine (Figure 1A, B). In parallel, a dose-dependent decrease of IκBα levels in cytosolic fractions of macrophages (Figure 1C, D) was found following exposure to norepinephrine or epinephrine. Similar results were obtained with mouse neutrophils (data not shown).

**Figure 2 pone-0004414-g002:**
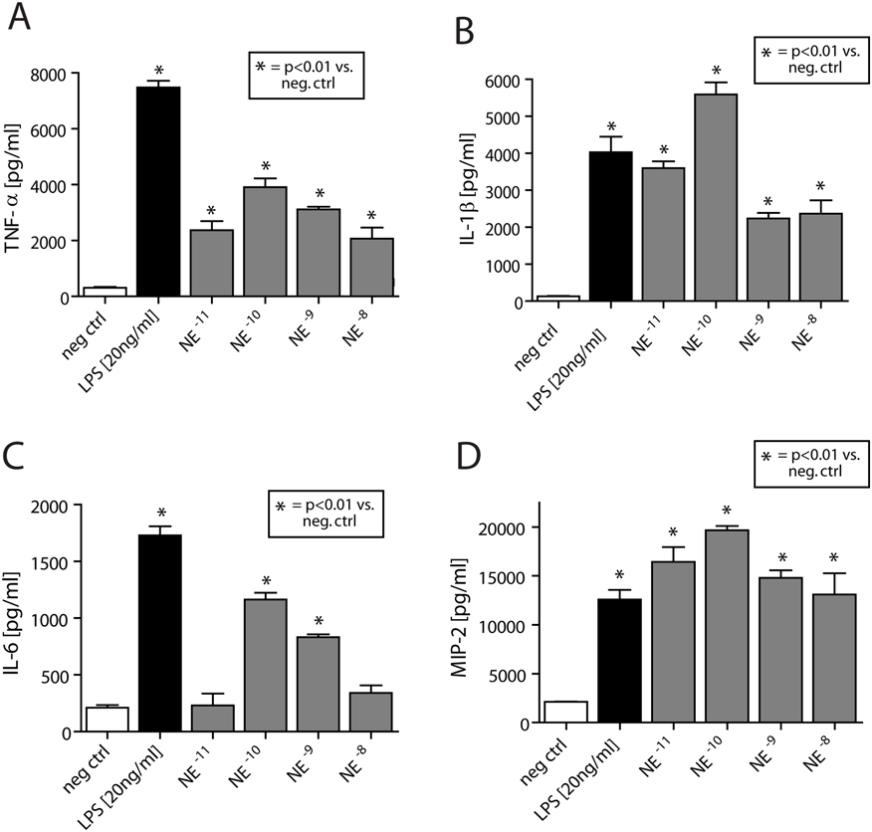
Isolated peritoneal mouse macrophages were exposed to various concentrations of norepinephrine (10^−11^–10^−8^ M) or LPS (20 ng/ml, positive control) for 30 min at 37°C. Then, supernatant fluids were obtained and subjected to ELISA analysis for TNF-α (A), IL-1β (B), IL-6 (C) and MIP-2 (D). Each bar represents n = 4–7. Neg ctrl, incubation with HBSS.

**Figure 3:**
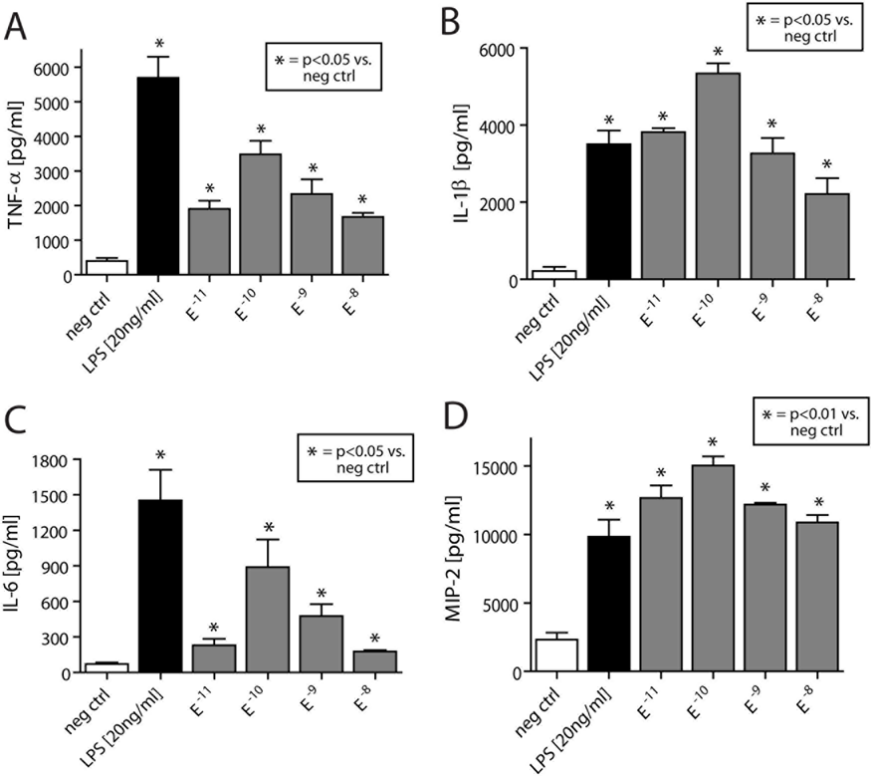
Following isolation, peritoneal mouse macrophages incubated to HBSS (negative conrol), LPS (20 ng/ml, positive control) or 10^−11^–10^−8^ M epinephrine (30 min, 37°C). Obtained supernatants were analyzed for TNF-α (A), IL-1β (B), IL-6 (C) and MIP-2 (D) using ELISA measurements. n = 4–7 per experimental group.

This suggests induction of intracellular proinflammatory pathways by norepinephrine and epinephrine. To evaluate if this catecholamine-induced activation of NFκB was followed by increased downstream production of proinflammatory cytokines, peritoneal mouse macrophages were incubated for 4 hrs at 37°C with either HBSS (negative control), LPS (positive control, 20 ng/ml), or various doses of norepinephrine or epinephrine that lead to NFκB activation (10^−11^–10^−8^ M). The cell supernatant fluids were evaluated for TNFα, IL-1β and IL-6 and MIP-2 by ELISA. Exposure to norepinephrine or epinephrine not only induced activation of NFκB (Figure 1), but caused release of TNFα, IL-1β and IL-6 and MIP-2 from isolated macrophages in a dose dependent manner (Figures 2 and 3). Interestingly, 10^−10^ M norepinephrine or epinephrine alone caused ≥50% of the amounts of released cytokines when compared to macrophages incubated with LPS (20 ng/ml).

### Increased Baseline-Expression of Catecholamine-generating Enzymes and Upregulation of Catecholamine Secretion in Phagocytes obtained from Adrenalectomized (ADX) Animals

**Figure 4:**
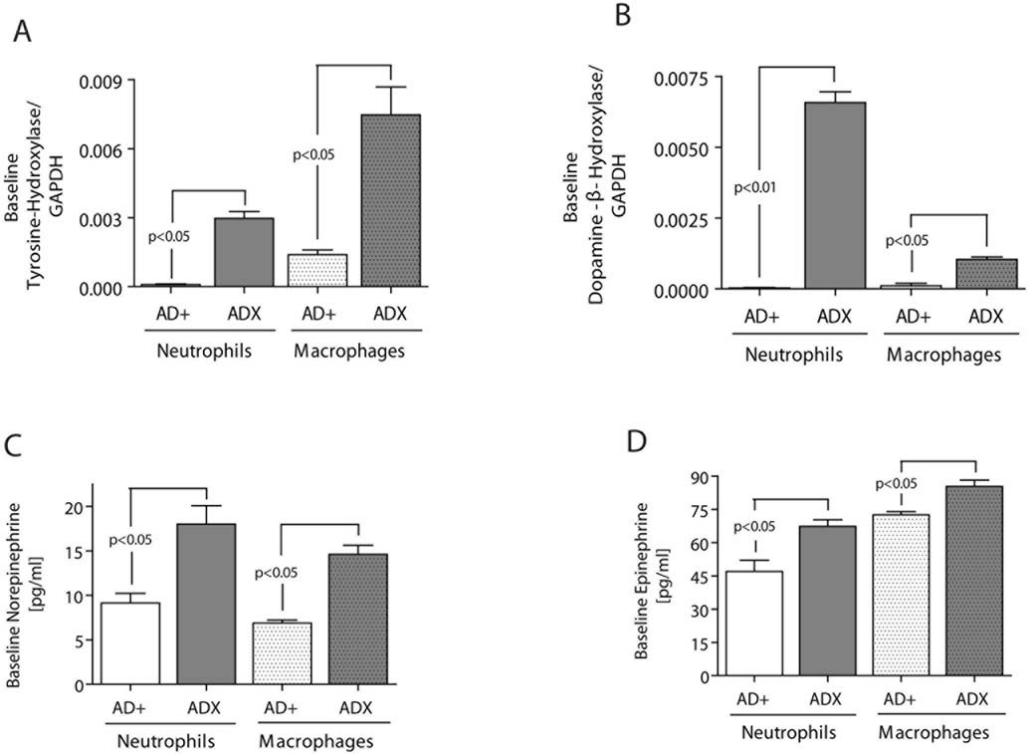
Rat blood neutrophils and rat alveolar macrophages were obtained from AD+ and ADX animals and mRNA isolated and subjected to real-time PCR analysis for both key-enzymes of catecholamine synthesis, tyrosine-hydroxylase (rate limiting step) and dopamine-β-hydroxylase (final conversion step) (A, B). Following 15 min culture of unstimulated phagocytes derived from AD+ and ADX animals, supernatant fluids were analyzed for norepinephrine (C) and epinephrine (D) by ELISA. Each bar n = 5.

We have recently identified phagocytes as a newly recognized source of norepinephrine and epinephrine [8]. To investigate the inflammatory potential of phagocyte-derived catecholamines, we isolated both blood neutrophils and alveolar macrophages from otherwise healthy and untreated adrenal-intact (AD+) and adrenalectomized (ADX) rats. When mRNA was obtained from isolated, unstimulated cells, phagocytes from ADX animals expressed higher baseline levels for the two key enzymes involved in catecholamine synthesis, tyrosine-hydroxylase (TH) and dopamine-β-hydroxylase (DBH) (Figure 4A, B), as demonstrated by real-time PCR. In parallel, the basal secretion (after 15 min in culture) of norepinephrine and epinephrine from isolated and otherwise untreated blood neutrophils and alveolar macrophages was significantly increased in phagocytic cells derived from ADX animals when compared to cells from AD+ animals (Figure 4C, D). This may represent a compensatory response in order to maintain intrinsic catecholamine levels in the absence of adrenal glands.

### Adrenalectomized Animals Display Elevated Baseline Levels in Plasma of Proinflammatory Mediators

To investigate whether the increased phagocytic baseline production of catecholamines in ADX rats (Figure 4) would result in activation of NFκB (Figure 1) with subsequent release of TNFα, IL-1β and IL-6 and MIP-2 by macrophages (Figures 2 and 3) *in vivo*, plasma from healthy and otherwise untreated AD+ or ADX animals was obtained and screened for baseline levels of proinflammatory mediators. As shown in Table 1, ADX animals displayed but statistically significant increase in plasma levels of the proinflammatory cytokines (TNFα, IL-1β and IL-6), as well as the inflammatory chemokine, CINC-1, when compared to AD+ littermates.

**Table 1:** Plasma Baseline Levels of Proinflammatory Mediators (pg/ml).

Mediators	AD+	ADX
TNFα	26±1	34±2*
IL-1β	44±4	61±2*
IL-6	105±4	128±1*
CINC-1	23±1	27±1*

For each sample, n = 8–10.

Data are expressed as mean±SEM.

* = p<0.01 vs. AD+.

### Adrenalectomy Exacerbates Acute Lung Injury

**Figure 5:**
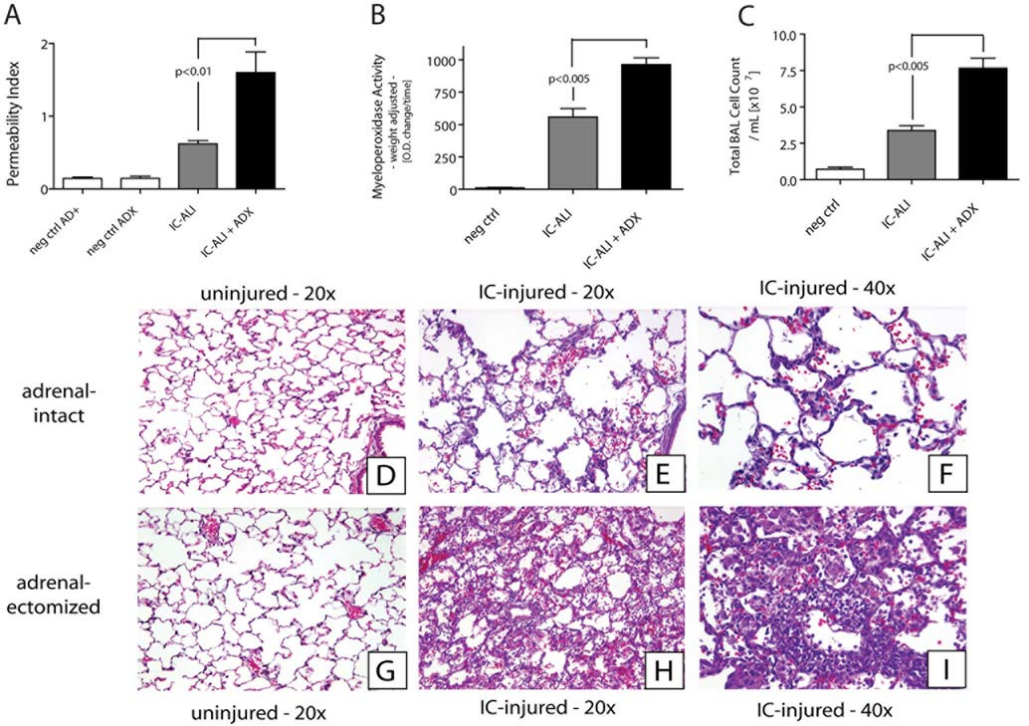
IgG immune complex (IC)-induced lung injury was induced in adrenal-intact (AD+) and adrenalectomized (ADX) rats. Vascular leakage (A), content of myeloperoxidase in lung extracts (B) and total white cell content in BAL fluids (C) were assessed. In negative controls, intratracheal administration of anti-BSA was replaced by PBS. Lungs were surgically removed 4 hr after intrapulmonary deposition of IgG immune complexes, tissues fixed in buffered 5% formaldehyde, and paraffin-embedded lung sections were stained with hematoxylin and eosin (D–I). Frames D–F, histology of lungs from adrenal-intact animals; frames G–I represent lungs from adrenalectomized littermates. Sections are representative for at least three rats per group. For each bar n>5 rats.

**Figure 6:**
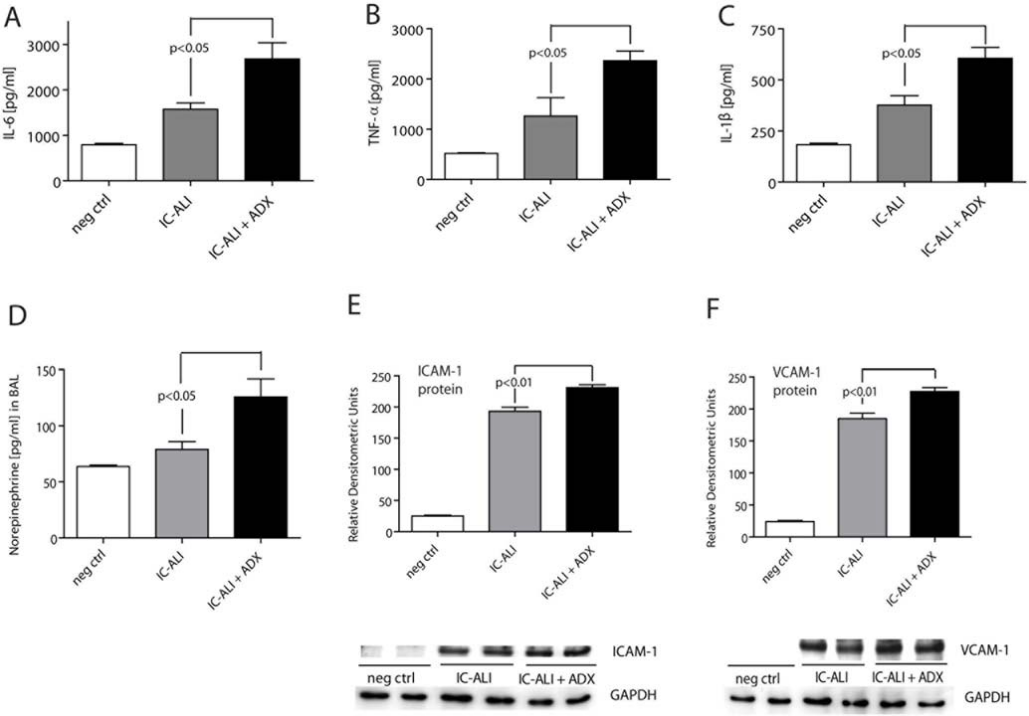
BAL fluids 4 hr after immune complex-induced lung injury, showing content of interleukin-6 (IL-6) (A), tumor necrosis factor α (TNFα) (B), interleukin-1β (IL-1β) (C) and norepinephrine (D). Four hours after induction of injury, whole lungs were flushed, surgically removed, homogenized and subjected to Western blot analysis for the adhesion molecules ICAM-1 (E) and VCAM-1 (F). For Western blot studies, experiments were repeated, using 5 different samples per group. Blots shown are of representative bands. For each bar n≥5 rats. Abbreviations: neg ctrl, negative control; AD+, adrenal-intact animals; ADX, adrenalectomized animals; IC-ALI, immune complex-induced acute lung injury.

Figures 5 and 6 display data obtained in the immune complex-induced ALI model. As shown in Figure 5, adrenalectomized (ADX) rats showed a greatly enhanced intensity of the acute inflammatory response. The permeability index (an indicator of vascular leakage of albumin) increased nearly 3 fold in ADX rats (Figure 5A) when compared to AD+ rats. Myeloperoxidase (MPO) content in lung homogenates increased nearly 50% in ADX rats (Figure 5B), and bronchoalveolar lavage (BAL) fluids had a higher leukocyte content (by nearly 2 fold), due to increased content of PMNs which accounted for >95% of BAL leukocytes (Figure 5C). The intensified level of injury was also apparent in morphological changes in lungs (Figure 5, frames D–I). While ADX and adrenal-intact rats exhibited comparable features in control (uninjured) lungs (Figure 5D, G), ADX animals had clearly increased evidence of injury as reflected in intensified number of PMNs, fibrin and hemorrhage in the interstitial and alveolar compartments (Figure 3H, I) when compared to lungs from adrenal intact rats (Figure 5E, F).

Enhanced lung injury in ADX rats was also reflected in BAL fluid analysis from adrenal-intact and ADX rats, which consistently showed significantly increased levels of proinflammatory cytokines (IL-6, TNFα and IL-1β) (Figure 6A–C) in ADX animals. In parallel, the levels of norepinephrine in BAL fluids from injured lungs were significantly elevated in ADX animals when compared to adrenal-intact littermates (Figure 6D). As demonstrated by Western blots in lung homogenates, the adhesion molecules ICAM-1 and VCAM-1 were modestly and significantly upregulated during lung injury (Figure 6, lower frames E, F). ADX animals had higher levels of these cell adhesion molecules than their adrenal-intact littermates. Finally, as expected, ADX rats showed evidence suggesting enhanced activation of NFκB in lung homogenates as assessed by EMSA (data not shown).

**Figure 7:**
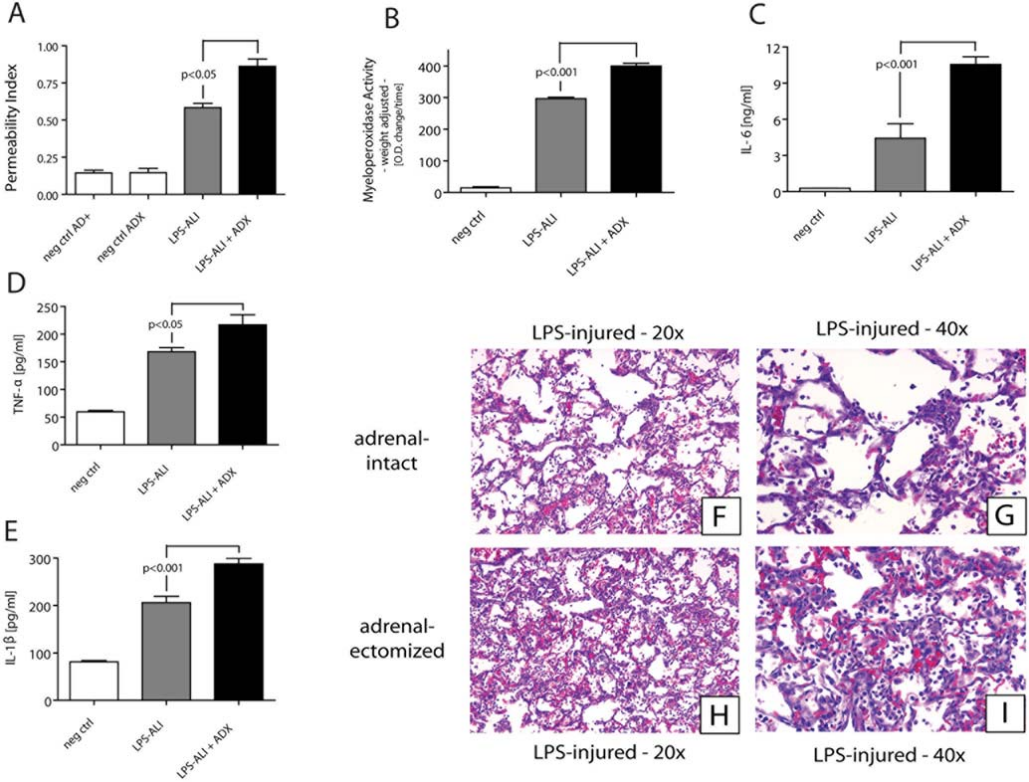
ALI was induced by airway instillation of LPS (300 µg). In parallel to immune complex-induced ALI, vascular leakage (A) and myeloperoxidase (MPO) activity in lung extracts (B) 6 hr after initiation of injury were measured. Bronchioalveolar lavage fluids obtained 6 hr after introduction of LPS-induced lung injury were assessed for content of IL-6 (C), TNFα (D), and IL-1β (E). Following surgical removal 6 hr after intrapulmonary LPS deposition, lungs were formalin-fixed, paraffin-embedded and stained with hematoxylin and eosin (F–I). Insets F and G show lungs of adrenal-intact animals, while lungs of rats lacking their adrenal glands are presented in insets H and I. For each bar n>5 rats. Abbreviations used: neg ctrl, negative control; AD+, adrenal-intact animals; ADX, adrenalectomized animals; LPS-ALI, LPS-induced acute lung injury.

To exclude that these results were unique to immune complex-induced ALI, lung injury was also induced in rats by intra-tracheal administration of LPS and subjected to analysis for vascular leakage, MPO content, BAL fluid cytokines measurements and histological analysis. The results, as shown in Figure 7, paralleled the pattern found in IC-induced lung injury, indicating amplified injury in ADX rats as defined by increased albumin leakage (frame A), elevated MPO content (frame B), amplified cytokine levels in BAL fluids (frames C–E) and enhanced histopathology of inflammation (neutrophils, fibrin) and injury (hemorrhage) (frames F–I).

### The α_2_-adrenergic Receptor Mediates the Severity of Acute Lung Injury in ADX Rats

**Figure 8:**
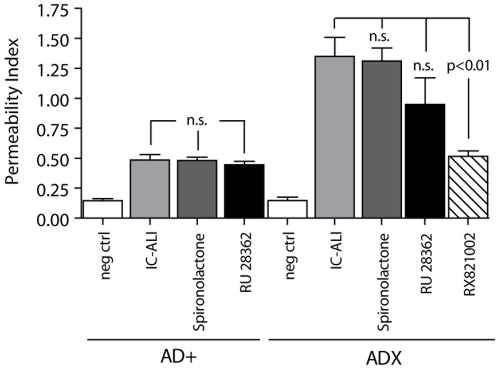
Severity of IgG-IC ALI following pharmacologic blockade of mineralocorticoid and glucocorticoid receptors (by spironolactone and RU 28362, respectively) in adrenal-intact and ADX animals, as assessed by permeability index. The α_2_-adrenergic antagonist RX 821002 was employed in ADX rats. Each bar represents n≥5 rats. Neg ctrl, negative control with intratracheal instillation of PBS.

Recently, we have described a central role for α_2_-adrenoceptor in the pathophysiology of experimental acute lung injury [8]. Pharmacological α_2_-adrenoceptor blockade by RX821002 greatly reduced the intensity of ALI, while blockade of all other adrenoceptors failed to change the intensity of lung injury [8]. To determine if α_2_-adrenoceptors or rather the absence of mineralocorticoids and glucocorticoids might account for the increased level of inflammatory lung injury in ADX rats, we administered the specific α_2_-adrenoceptor blocker, (RX 821002), or the competitive aldosterone receptor antagonist (spironolactone) in adrenal-intact and adrenalectomized animals immediately after induction of IC-ALI. The glucocorticoid receptor antagonist, RU 28362, was administered 1 hr before initiation of lung injury. As shown in Figure 8, blockade of the α_2_-adrenergic receptor significantly reduced by 70% (p<0.01) the intensity of the albumin leak into lungs of ADX rats. In AD+ animals, blockade of the α_2_-adrenoceptor reduced the albumin leak following ALI by ∼46%, as reported earlier [8]. In contrast, glucocorticoid or mineralocorticoid receptor blockade (by RU 28362 and spironolactone, respectively) neither exacerbated nor reduced the level of injury in both adrenal-intact or ADX animals (Figure 8), suggesting that it is adrenergic receptor engagement rather than cortical mineralocorticoid or glucocorticoid receptor engagement that affects the inflammatory response in ADX animals.

## Discussion

There is increasing evidence that the immune system and the central nervous system (CNS) interact to modulate each other via autonomic pathways [2]–[5]. Immune mediators and cytokines released by the innate immune system rapidly activate neuronal responses, resulting in amplified local immune responses designed to clear pathogens and triggering regional neural and systemic neuroendocrine responses, (including local catecholamine production and systemic glucocorticoid release), both of which seek to return the system to a homeostatic state [1].

Following interaction with adrenergic receptors, the physiological actions of catecholamines can be terminated by cellular reuptake, followed by their intracellular inactivation by monoamine oxidase (MAO) or catechol-*O*-methyltransferase (COMT). Moreover, there is now evidence for the presence of dopamine and norepinephrine transporters (DAT and NAT, respectively) on lymphocytes, which facilitate the rapid local removal of dopamine or norepinephrine by reuptake [24]–[27]. Similarly, catecholamine-specific transporters have been described on nuclear membranes of lymphocytes, which actively transport catecholamines from the cytoplasm into the cell nucleus, where catecholamines can interact with nuclear receptors and regulate proliferation or apoptosis [28]–[31]. Since mitochondria-associated MAO and the cytosolic COMT do not enter the nucleus, it remains to be determined how and by which mechanism intranuclear actions of catecholamines are terminated. In the present study we describe in macrophages a catecholamine-induced, dose dependent activation of NFκB p65 and a decrease of cytosolic IκBα (Figure 1). A similar pattern has been described when alveolar macrophages were exposed *in vitro* to IgG immune complexes [32]. Thus, catecholamines enhance cytokine release by macrophages (Figures 2 and 3) via activation and translocation of NFκB (Figure 1), indicating that catecholamines are powerful cellular hormones that self-regulate the activation level and the inflammatory potential of inflammatory cells.

It is well established that phagocytes (PMNs and alveolar macrophages) are essential for initiation of acute lung injury in our present models [33]–[36]. These cells are known to express all adrenergic receptors (both α and β subtypes) and to produce enhanced levels of TNFα in the presence of an α_2_-adrenergic agonist [8], [19], [20]. Accordingly, in the present study, the mechanism of augmented injury in ADX rodents might be related to an elevated production of phagocyte-derived catecholamines in an attempt to restore systemic catecholamine levels in the absence of the adrenal glands, resulting in increased catecholamine production by PMNs and macrophages, as suggested in Figure 4. This seems to be followed by direct activation of NFκB and “priming” of macrophages, leading to an increased baseline production of proinflammatory mediators (Figures 1–3 and Table 1). Upon a “second hit”, such as exposure to LPS, IgG-IC or other inflammatory stimuli, the inflammatory response is greatly accentuated. Another possibility might be a compensatory over-activity of pulmonary sympathetic nerve endings or increased catecholamine production by lymphocytes, resulting in increased norepinephrine levels in BAL fluids. However, we recently demonstrated in the present model of ALI that neither T cells nor sympathetic nerves (by cell depletion or chemical sympathectomy, respectively) are involved in events leading to ALI, but, rather, alveolar macrophages and neutrophils are responsible for increased catecholamine levels in BAL fluids following IC-ALI [8]. Moreover, in a recent study, untreated and healthy bilaterally adrenalectomized rats displayed morphological signs of renal inflammation when compared to untreated adrenal-intact littermates [37], confirming our findings that adrenalectomized rats exhibit a certain proinflammatory priming. Thus, it is now becoming evident, that the sympathetic nervous system may play a dualistic role during the inflammatory response, than previously thought. While it clearly has profound anti-inflammatory effects during *systemic* inflammation as described above [1], [3], we are now beginning to understand that the *local* inflammatory response can be immensely boosted through local, cell-dervied catecholamine production and subsequent adrenergic signaling in various immune cells [7], [8].

Further hormonal key players antagonizing inflammation are glucocorticoids, which are rapidly released from the adrenal cortex following activation of the HPA axis and down-regulate inflammation [38]. It was somewhat surprising that greatly increased levels of ALI occurred in ADX rats and that this was not related to engagement of mineralocorticoid or glucocorticoid receptors (Figure 8). Glucocorticoids ultimately inhibit transcription, which takes up to several hours, whereas lung injury in the current model peaks 4 or 6 hr after initiation. Thus, it seems unlikely that various glucocorticoid effects could have been fully developed in this short period of time. Moreover, rats possess the ability to synthesize steroids in non-adrenal tissues. The rat CNS has been identified as a source of 11β-hydroxylase and aldosterone synthase [22], [39], [40]. Other non-adrenal sources include the kidney and vascular tissues [40]. Thus, it seems likely that, in the absence of the adrenal glands, these extra-adrenal sources of corticosterone and aldosterone compensate for the lack of the adrenal medulla in an attempt to maintain systemic levels of corticoids.

This study suggests that catecholamines activate macrophage NFκB with subsequent cytokine production in a dose dependent manner. Upregulation of phagocyte-derived catecholamines (by adrenalectomy) results in intensification of the acute inflammatory response.

## Materials and Methods

### Reagents

Norepinephrine and epinephrine were obtained from Sigma-Aldrich (St. Louis, MO) and were of highest purity. These chemical compounds were synthetically manufactured under sterile conditions and were thus virtually free of endotoxin, RNA or DNA according to the manufacturer.

### Animals and Anaesthesia

All investigative procedures and the animal facilities conformed to the Guide of Care and Use of Laboratory Animals published by the US National Institutes of Health. The study was approved by the University Animal Care and Use Committee (UCUCA) and performed according to appropriate guidelines. Specific pathogen-free male C57BL/6 mice (Jackson Laboratories, Bar Harbor, ME) were used for data displayed in Figure 1 and Table 1. Adrenalectomized Long-Evans rats (300–325 g) were obtained from Taconic, Hudson, NY. Un-manipulated, adrenal-intact littermates (300–325 g; Taconic, Hudson, NY) served as controls.

### Isolation of mouse macrophages

Peritoneal mouse macrophages were obtained using the thioglycollate method. Mice were injected with 1.5 ml of 2.4% thioglycollate in ddH_2_O. Four days later, transmigrated macrophages were harvested by instillation and aspiration of 8 ml PBS (Gibco, Grand Island, NY). Cells were then spun down and subjected to an additional washing step using PBS. Macrophages were then resuspended in HBSS (with Ca^2+^/Mg^2+^). The obtained cell suspension was of high purity, as determined by optical cell differential counts (neutrophils: 0%, macrophages: 97%, lymphocytes: 1%, eosinophils: 2%). Mouse macrophages were exclusively used for the experiments in Figures 1 through [Fig pone-0004414-g002] 3, since high cell numbers were needed for these experiments in order to obtain adequate numbers of nuclei. For all other experiments, rat phagocytes were used.

### Isolation of rat neutrophils and macrophages

For rat neutrophil isolation, whole blood samples were drawn from the inferior vena cava into syringes containing anticoagulant ACD (Baxter Health Care, Deerfield, IL, USA) (0.1 ml/ml blood). Cells were isolated by using Ficoll-Paque gradient centrifugation (Pharmacia Biotech AB, Uppsala, Sweden) followed by a dextran sedimentation step. After hypotonic lysis of residual blood cells, neutrophils were resuspended in HBSS. The purity of this neutrophil suspension was >95%. Alveolar macrophages were obtained by bronchioalveolar lavage of rats by instilling and withdrawing 10 ml sterile Dulbecco's PBS (without Ca^2+^/Mg^2+^) three times from the lungs via an intratracheal cannula. Cells were then spun down and resuspended in HBSS. The purity of the cell suspension was >95%, with the rest of the cells being lymphocytes.

### Measurement of NFκB activation

Isolated mouse neutrophils were adjusted to 10×10^6^/ml and resuspended in RPMI medium containing 0.5% BSA (Sigma-Aldrich, St. Louis, MO). Cells were then allowed to settle down for 2 hrs at 37°C/5%CO_2_ to discard non-adherent and non-viable cells. Upon stimulation with various doses of norepinephrine or epinephrine (10^−12^–10^−6^ M; both Sigma-Aldrich, St. Louis, MO) for 30 min at 37°C/5%CO_2_, nuclear extracts were obtained using a commercially available kit (“Nuclear Extract Kit”, Active Motif, Carlsbad, CA) according to the manufacturer's instructions, quantified by BCA™ protein measurement (Thermo Scientific, Rockford, IL), protein adjusted and stored at −80°C until further analysis. NFκB p65 activation was assessed with an ELISA kit (“TransAM™ NFκB p65”, Active Motif, Carlsbad, CA) according to the manufacturer's instructions. Recombinant NFκB p65 protein (Active Motif, Carlsbad, CA) was used to generate the standard curve.

### Western Blot analysis for IκBα

Following isolation and incubation with various concentrations of norepinephrine or epinephrine (30 min, 37°C), peritoneal mouse neutrophils (5×10^6^/ml) were lysed and cytosol was subjected to Western Blotting analysis for IκBα. Samples were separated in a denaturing polyacrylamide gel and transferred to a PVDF membrane. After blocking with 5% milk-TBST washing in TBST, membranes were then incubated in the appropriate primary antibodies (IκBα or GAPDH; both from Abcam, Cambridge, MA) at 4°C overnight. After washing, membranes were incubated with the appropriate HRP-conjugated secondary antibodies (Amersham, Arlington Heights, IL) and analyzed by ECL development. Neg ctrl, incubation with HBSS.

### Cytokine-production by neutrophils following norepinephrine and epinephrine incubation

Isolated mouse neutrophils were adjusted to 5×10^6^ cells/ml and incubated with HBSS (neg ctrl), 20 ng/ml LPS (pos ctrl), norepinephrine (10^−10^ M) or epinephrine (10^−10^ M) for 4 hrs at 37°C/5%CO_2_. Supernatants were analyzed by ELISA for TNFα, IL-1β, IL-6 and MIP-2 (all R&D Systems, Minneapolis, MN).

### Isolation of total RNA and detection of rat tyrosine-hydroxylase (TH) and rat dopamine-β-hydroxylase (DBH) by real-time quantitative PCR analysis

Total RNA was extracted from isolated phagocytes using Trizol reagent (Life Technologies, Grand Island, NY) according to the manufacturer's instructions. Reverse transcription was performed with 1 µg RNA using Reverse Transcriptase AMV (Roche, Indianapolis, IN) according to the manufacturer's instructions. Real-time quantitative PCR was then performed using SYBR® Green PCR Master Mix (Applied Biosystems, Foster City, CA). The following primers were used: rat TH: FOR 5′-AGT CTG GCC TTC CGC GTG TTT CAA-3′ and REV 5′-GGG CGC TGG ATA CGA GAG GCA TAG-3′; rat DBH FOR: 5′-CTG CGA CCC CAA GGA TTA TG-3′ and REV 5′-CAG CAC GTG GCG ACA GTA GTT-3′; rat GAPDH: FOR 5′-CGG CAA GTT CAA CGG CAC AGT CA-3′ and REV 5′-CTT TCC AGA GGG GCC ATC CAC AG-3′. Product sizes were 458 bp (TH), 439 bp (DBH), and 424 bp (GAPDH).

### Analysis of cell supernatants for catecholamines

EIA kits for Norepinephrine and Epinephrine were obtained from Rocky Mountain Diagnostics, Colorado Springs, CO. Supernatant fluids from phagocytic cells were analyzed according to the manufacturer's instructions.

### IgG immune complex and LPS-induced alveolitis

Intraperitoneal ketamine (100 mg/kg body weight) (Fort Dodge Animal Health, Fort Dodge, IA) was used for anesthesia and intraperitoneal xylazine (13 mg/kg body weight) (Bayer Corp. Shawnee Mission, KS) for sedation. Induction of IgG immune complex-induced alveolitis was performed as previously described [41]. Rats received 2.5 mg of rabbit polyclonal IgG anti-BSA (ICN Biomedicals, Costa Mesa, CA) in 300 µl of PBS intratracheally, followed by i.v. injection of either 10 mg of BSA in 0.5 ml PBS (injury) or 0.5 ml PBS (neg ctrl). For LPS-induced alveolitis, rats received 300 µg of LPS (Sigma Aldrich, St. Louis, MO) in a total volume of 300 µl PBS intratracheally. Maximum level of injury in LPS-alveolitis was reached 6 hr after LPS-instillation. Permeability index was performed as described [41] and quantified by calculating the amount of radioactivity in lungs divided by the amount of radioactivity in 1.0 ml blood.

### Lung myeloperoxidase content

Whole-lung MPO activity was quantified as previously described [42].

### BAL fluid analysis

BAL fluids were collected by instilling and withdrawing 10 mL of sterile PBS three times from rat lungs. Cellular contents were counted by Coulter cytometry after lysis of erythrocytes. Contents of interleukin IL-6, TNFα, IL-1β and noradrenaline in BAL fluids were measured using ELISA kits (R&D Systems, Minneapolis, MN and Rocky Mountain Diagnostics, Colorado Springs, CO, respectively).

### Morphological assessment of lung injury

Rat lungs were fixed by intratracheal instillation of 10 ml buffered (pH 7.2) formalin (10%). The lungs were further fixed in a 10% buffered formalin solution for histological examination by tissue sectioning and staining with H&E.

### Western blot analysis for ICAM-1 and VCAM-1

Flushed whole rat lungs were homogenized in ice-cold RIPA buffer containing a protease-inhibitor cocktail (Roche, Indianapolis, IN). After protein extraction, sonication and protein measurement, samples were separated in a denaturing polyacrylamide gel and transferred to a PVDF membrane. After blocking with 5% milk-TBST washing in TBST, membranes were then incubated in appropriate primary antibodies (ICAM-1, VCAM-1: Santa Cruz Biotechnology, Santa Cruz, CA; GAPDH: Abcam, Cambridge, MA) at 4°C overnight. After washing, membranes were incubated with the appropriate HRP-conjugated secondary antibodies (Amersham, Arlington Heights, IL) and analyzed by ECL development.

### Receptor agonists and antagonists

Agonists and antagonists (all Sigma Aldrich, St. Louis, MO) were administered to rats intraperitoneally in a total volume of 1.5 ml. Following agents were used: 1,2-propanediol (vehicle, 1 ml/kg), RX 821002 (α_2_-adrenoceptor antagonist, 2.5 mg/kg), RU 28362 (glucocorticoid receptor antagonist, 150 µg/kg) and spironolactone (mineralocorticoid receptor antagonist, 50 mg/kg). RX 821002 and spironolactone were administered immediately before induction of IC-induced lung injury, while RU 28362 was administered 1 hr before initiation of lung injury.

### Statistical analysis

All values are expressed as means±SEM. Data were analyzed with a one-way ANOVA and individual group means were then compared with a Student-Newman-Keuls test. Differences were considered significant when p≤0.05.
